# The Hertel classification can't predict the risk of humeral head osteonecrosis after osteosynthesis using an anterolateral approach

**DOI:** 10.1016/j.clinsp.2023.100173

**Published:** 2023-03-03

**Authors:** Márcio Alves Cruz, Guilherme Grisi Mouraria, Fernando Kenji Kikuta, Daniel Romano Zogbi, Sérgio de Paula Coelho, Maurício Etchebehere

**Affiliations:** Orthopedics and Traumatology, Universidade Estadual de Campinas (UNICAMP), Campinas, SP, Brazil

**Keywords:** Shoulder fracture, Osteonecrosis

## Abstract

**Background:**

Proximal humeral fractures can progress to osteonecrosis of the humeral head. Hertel developed a binary classification system (12 subtypes) and demonstrated that some patterns have more risk to develop osteonecrosis. Hertel described the prevalence and the risk factors for Humeral head osteonecrosis after osteosynthesis using a Deltopectoral approach. Few studies have evaluated the prevalence and the capacity of Hertel's classification to predict Humeral Head osteonecrosis following osteosynthesis of proximal Humeral fractures through the anterolateral approach. The objectives of this study were to correlate osteonecrosis predictors established by the Hertel classification with the risk of developing osteonecrosis and its prevalence after osteosynthesis using the anterolateral approach.

**Methods:**

This was a retrospective study of patients who underwent osteosynthesis of proximal humerus fractures using an anterolateral approach. Patients were divided into two groups: high risk for necrosis (group 1) and low risk for necrosis (group 2) according to Hertel's criteria. The overall prevalence of osteonecrosis and the prevalence in each group were calculated. A radiological examination was performed in the true anteroposterior (Grashey), scapular, and axillary views, before and after the operation (minimum 1 year after surgery). A Kaplan-Meier curve was used to assess the pattern of the temporal evolution of osteonecrosis. The groups were compared using the Chi-square test or Fisher's exact test. The unpaired *t*-test (parametric variables – age) and the Mann-Whitney test (non-parametric time between trauma and surgery) were used.

**Results:**

In total, 39 patients were evaluated. The postoperative follow-up time was 14.5 ± 3.3 months. The time to onset of necrosis was 14.1 ± 3.9 months. Sex, age, and time between trauma and surgery did not influence the risk of necrosis. Type 2, 9, 10, 11, and 12, or fractures with posteromedial head extension less than or equal to 8 mm, or diaphysis deviation greater than 2 mm, as well as grouping did not influence the risk for osteonecrosis.

**Conclusions:**

Hertel's criteria were not able to predict the development of osteonecrosis after osteosynthesis of proximal humerus fractures performed through the anterolateral approach. The total prevalence of osteonecrosis was 17.9% with a tendency toward an increased incidence after 1 year of surgical treatment.

## Highlights

• Proximal humeral fractures can progress to humeral head osteonecrosis
• It's controversial if the Hertel classification can predict humeral head osteonecrosis.
• Proximal Humeral Fractures can be fixed through the anterolateral approach.
• Objective: to correlate the Hertel's predictors with the humeral head osteonecrosis.
• Hertel's criteria were not able to predict the risk for humeral head osteonecrosis.
• The overall prevalence humeral head osteonecrosis was 17.9%.

## Introduction

Proximal humeral fractures can progress to Humeral Head Osteonecrosis (HHO) as a result of blood supply interruption caused by trauma.^1^ The anterior humeral circumflex artery is often damaged by trauma, so the posterior humeral circumflex artery is of great importance in maintaining vascularization of the humeral head.^2^ Fracture characteristics including comminution, involvement of the articular surfaces, and bone fragments in the posteromedial humerus, may increase the risk of HHO. Because of that, the rates of osteonecrosis can range from 0% to 75%.^3^, ^4^, ^5^

The risk of HHO is an important factor in surgical decision-making. Fractures with a high risk of necrosis (especially in the elderly) can be treated with arthroplasty. In contrast, young patients and/or low-risk fractures may be treated with osteosynthesis.^6^ Several authors have investigated fracture patterns and correlated them with the risk of HHO. In 1971, Neer observed that four-part fractures were associated with a greater risk of necrosis.^7^ More recently, Hertel developed a binary classification system (12 subtypes) and demonstrated that patterns 2, 9, 10, 11, and 12, and fractures with posteromedial head extension less than or equal to 8 mm, or diaphysis deviation greater than 2 mm (injury to the medial hinge), were at increased risk for HHO.^8^

It remains controversial whether the Hertel classification can predict HHO risk.^5^ In Hertel's study, humeral head perfusion was assessed intraoperatively through the deltopectoral approach.^8^

The period of time that the Proximal Humeral Head progress to osteonecrosis ranges from 6 months to 2 years and the diagnoses can be made using Radiographs. The use of Magnetic Resonance can identify osteonecrosis in the early stage.^1^

Osteosynthesis for proximal humerus fractures can be performed using either a Deltopectoral or Anterolateral approach. Both require muscle dissection and retraction for adequate lateral humerus exposure. The anterolateral approach favors reaching the lateral aspect of the humerus because it's not necessary to retract the Deltoid and Pectoral Major Muscles. Despite the fact that in anterolateral surgeons must dissect the axillar nerve, the incidence of nerve injury is relatively rare.^1^^,^^10^

Hertel described the incidence and the risk factors after osteosynthesis using a Deltopectoral approach. Few studies have evaluated the prevalence and the capacity of Hertl's classification to predict Humeral Head osteonecrosis following osteosynthesis of proximal Humeral fractures through the anterolateral approach.^8^

The Hypothesis is that Hertel's classification can predict the Risk of HHO after Osteosynthesis using Anterolateral Approach.

## Objectives

The primary objective of the study was to correlate osteonecrosis predictors, established by the Hertel classification, with the presence or absence of HHO following osteosynthesis of the proximal humerus through the anterolateral approach.

The secondary objective was to assess the prevalence of HHO after at least 1 year of postoperative follow-up.

## Materials and methods

This was a retrospective study of patients who underwent osteosynthesis for proximal humerus fractures via the anterolateral approach between 2016 and 2019.

Inclusion criteria: Patients underwent fracture osteosynthesis using the anterolateral approach and had all the radiological images required for preoperative Hertel classification and for HHO evaluation.

Exclusion criteria: Cases without complete documentation, fractures associated with a dislocation, and pathological fractures.

Patients which suffered fractures as a result of falls from weight as considered was considered low trauma energy. Motorcycles, bicycles, and accidents were considered high trauma energy.

The Hertel classification was used to classify fractures and stratify the risk of osteonecrosis. Radiographs were taken in anteroposterior, scapular, and axillary views. Preoperative investigations were evaluated by two examiners and the kappa test was used to assess agreement between examiners. The Hertel classification was used to divide the patients into two groups. Binary patterns 2, 9, 10, 11, and 12, or posteromedial head extension less than or equal to 8 mm, or diaphysis deviation greater than 2 mm (injury to the medial hinge), were allocated to group 1 (high risk for osteonecrosis). Fractures with binary patterns 1, 3, 4, 5, 6, 7, and 8, and/or posteromedial head extension greater than 8 mm, or diaphysis deviation less than 2 mm (medial hinge integrity) were allocated to group 2 (low-risk for necrosis).

Radiographic evaluations were performed using the SinapseR digital radiography program. Postoperative radiological examinations were performed at least 1 year after the surgical procedure. Osteonecrosis was identified by a radiologist. The presence of a cist, sclerotic changes in the Humeral Head, and subcondral collapse producing a crescent sign in Radiographs were considered positive for osteonecrosis.

Patients underwent surgery in a beach chair position. A 10-cm incision was made from the anterolateral edge of the acromion in a distal direction and parallel to the axis of the diaphysis. The anterior and middle portions of the deltoid were separated by blunt dissection and the axillary nerve was identified. After fracture reduction and fixation, a provisional plate with Kirschner wires (Humerus Gm-Reis^R^) was placed on the lateral face of the humerus below the anterior branch of the axillary nerve.

The total prevalence of HHO and the prevalence in each group were calculated. Comparisons between groups were performed using the Chi-Square or Fisher's exact tests. Non-categorical variables were tested for normality using the Kolmogorov-Smirnov test. The unpaired *t*-test (parametric variables) and the Mann-Whitney test (non-parametric) were also used. A Kaplan-Meier curve was used to assess the pattern of the temporal evolution of osteonecrosis. All analyzes were performed using the PASW statistics 27.0 software (IBM Corp., Armonk, NY, USA) adopting a significance level of 5%.

The research protocol was approved by the local ethics committee (Campinas State University ethics committee – registration n° 34384120.5.0000.540). All methods were analyzed by this committee and were carried out in accordance with relevant guidelines and regulations. Informed consent was obtained from all patients.

## Results

In total, 39 patients met the inclusion criteria, with a predominance of females (55.3%). The average age was 58.4 ± 12.0 years. The youngest patient was 21 and the older was 78 years old.

Patients who underwent surgery ranged from 1 to 15 days after the trauma. Postoperative follow-up time was 14.5 ± 3.3 months. Table 1 lists demographic data.

**Table 1:** Demographic data.

Variable	Value
Age (average ± SD), years	58.4 ± 12.0
Sex, n (%)	
Female	22 (56.4%)
Male	17 (43.6%)
Trauma-to-surgery days (average ± SD)	6.6 ± 4.4
Follow-up (average ± SD), months	14.5 ± 3.4

In terms of an agreement between observers, the lowest level was reached for the fracture extension criterion for the medial region of the head smaller than 8 mm (Kappa = 0.04; p = 0.60), while high levels of agreement were reached for medial hinge loss (Kappa = 0.79; p < 0.001) and binary classification (Kappa = 0.62; p < 0.001). To assess the risk for necrosis, the patients were grouped as high- and low-risk. After grouping, the agreement between the observers increased (Kappa = 0.86; p < 0.001). Table 2 lists the Kappa data.

**Table 2:** Interobserver kappa test for the Hertel classification.

Hertel's criteria	Kappa	p-value
Medial extension^a^	0.04	0.60
Medial hinge loss	0.79	<0.001
Binary classification	0.62	<0.001
High-risk group^b^	0.89	<0.001

a Fracture extension to head smaller than 8 mm.

b Risk of necrosis.

**Fig. 1:**
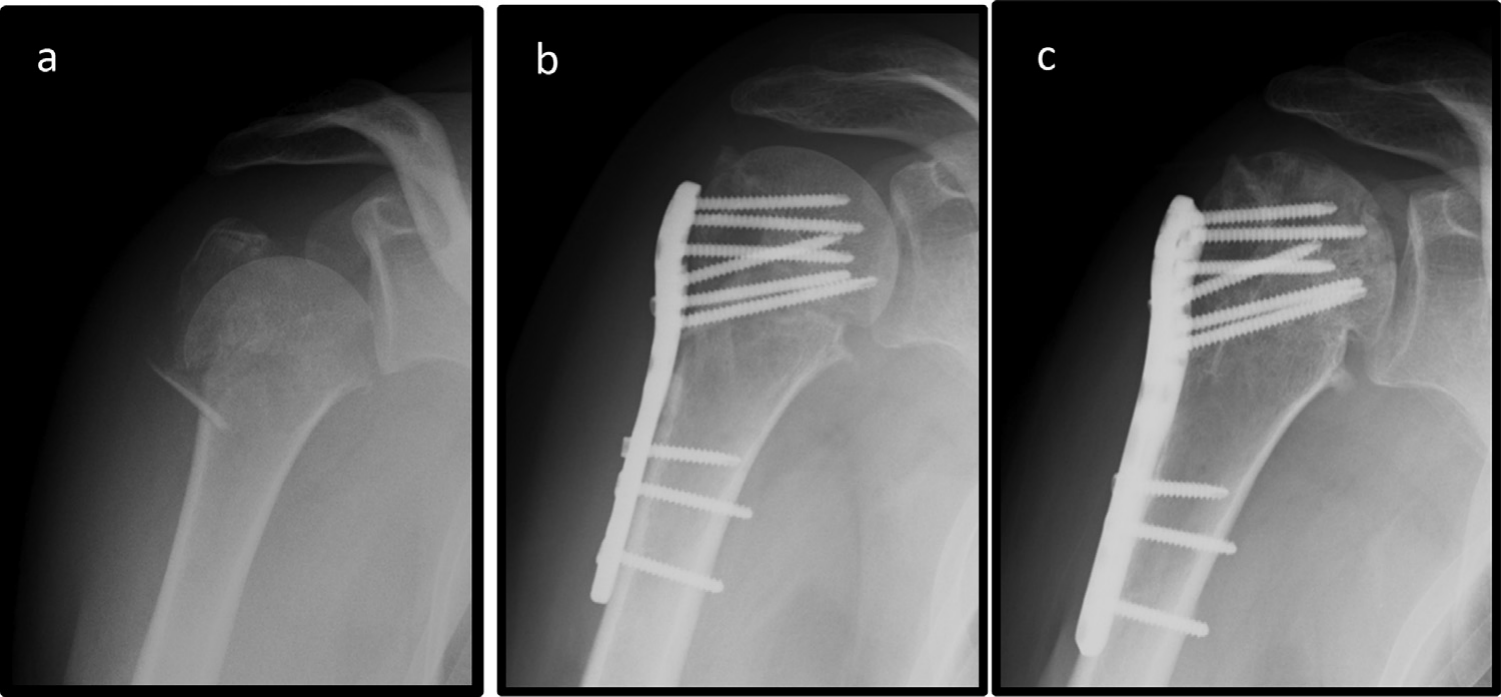
Radiographs showing initial fracture (a); one day after the surgery (b); six months after the surgery with radiological evidence of HHO (c).

Humeral head evolves to osteonecrosis in 7 (17.9%) patients. The mean time to onset of necrosis was 14.1 ± 3.9 months (range: 3–18 months). Two patients developed humeral head necrosis (Fig. 1) without collapse, while 5 patients had a collapse. Screw migration due to necrosis occurred in 3 patients and the synthesis material had to be removed.

**Fig. 2:**
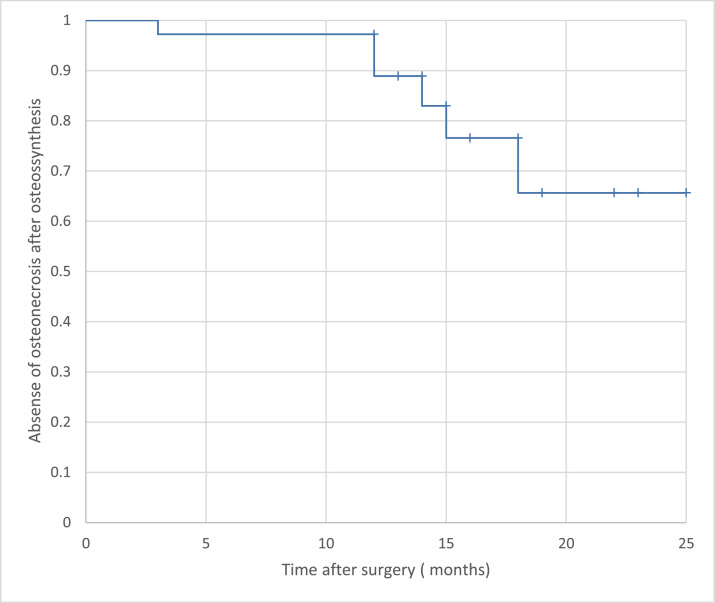
Kaplan-Meier curve with temporal evolution of osteonecrosis.

The Kaplan-Meier curve showed a tendency toward an increased incidence of HHO after 1 year of surgical treatment (Fig. 2).

The authors evaluated associations between factors including gender, age, trauma energy, and time elapsed after fracturing with HHO and found no significant associations (Table 3). The most common binary classification was the type 12 pattern (Table 4). Binary patterns and additional Hertel criteria influenced the development of HHO (Tables 4 and 5).

**Table 3:** Correlations between variables and osteonecrosis.

Variable	Presence of osteonecrosis	Absence of osteonecrosis	p-value
Trauma Energy, n (%)			1.00^a^
High	2 (5.1%)	10 (25.6%)	
Low	5 (12.8%)	22 (56.7%)	
Sex, n (%)			0.20^a^
Female	2 (5.1%)	20 (51.2%)	
Male	5 (12.8%)	12 (30.7%)	
Age (average/SD)	53.8 ± 17.7	59.4 ± 11.3	0.27^b^
Trauma-to-surgery (Median/min–max), days	8 (1–18)	6 (1–16)	1.0^a^

a Fisher's exact test.

b Student's *t*-test. ^c^Mann-Whitney *U* test (in days).

**Table 4:** Binary Hertel classification and association with osteonecrosis.

Binary pattern	Presence of osteonecrosis (n)	Absence of osteonecrosis (n)	p-value^a^
1	1	11	0.40
7	1	8	1.00
8	1	1	0.33
12	4	12	0.41

a Fisher's exact test; (n) total number of patients.

**Table 5:** Additional Hertel's criteria and association with osteonecrosis.

Variable	Presence of osteonecrosis	Absence of osteonecrosis	p-value
Medial hinge lesion, n (%)			0.69^a^
No	3 (7.7%)	17 (43.6%)	
Yes	4 (10.3%)	15 (38.4%)	
Head extension <8 mm, n (%)			0.52^a^
No	5 (12.8%)	25 (64.1%)	
Yes	2 (5.1%)	7 (18%)	

a Fisher's exact test; (n) total number of patients.

On the basis of the factors that could increase the risk for HHO, the authors divided the sample into two groups. However, the groups did not differ in terms of HHO development (Table 6). Three cases had 3 risk factors for osteonecrosis and none of them developed this complication.

**Table 6:** Development of osteonecrosis in the two groups.

Group	Presence of osteonecrosis, n (%)	Absence of osteonecrosis, n (%)	p-value^a^
Group 1	5 (12.8%)	19 (48.7%)	
Group 2	2 (5.1%)	13 (33.3%)	0.68
Total	7 (17.9%)	32 (82.1%)	

a Fisher's exact test; (n) total number of patients.

## Discussion

Humeral head osteonecrosis is one of the most frequent complications following osteosynthesis of proximal humerus fractures.^11^ In this study, the authors observed a prevalence of 17.9%, similar to that reported by Greiner et al., who evaluated a similar cohort.^12^ The prevalence of HHO after osteosynthesis ranges from 4%–30%.^11^

In terms of demographic characteristics, the authors observed a higher prevalence of fractures in females (56.4%) and the elderly (58.4 ± 12 years), which is in agreement with the literature.^13^ The authors did not find any influence of sex and age on the development of HHO.^11^

The time duration between trauma and surgery was longer in patients who developed HHO. However, it did not influence the development of osteonecrosis, which is also in agreement with the literature.^12^ Despite some controversy, most clinicians agree that the posterior circumflex artery provides the main arterial supply to the proximal end of the humerus after a fracture.^2^ It is possible that preservation of the posterior circumflex artery reduces the risk of HHO, and that the time duration between the trauma and the surgery does not influence the biological viability of the humeral head. The authors always avoid extending the dissection and muscle retractions in order to decrease the risk of arterial damage and a chance of HHO.

Some authors have investigated and classified fracture patterns. Hertel developed a classification system with criteria to predict HHO risk.^8^ The authors found a high concordance of the Hertel classification – Kappa = 0.62; p ≤ 0.001 (except for the posteromedial head extension criterion less than or equal to 8 mm – Kappa = 0.04; p = 0.6) between observers, as described in the literature.^14^

Despite the high level of agreement in terms of the Hertel classification and criteria between observers, the system was not able to predict the risk for HHO. Even after grouping the factors, there was no difference in the incidence of HHO between the groups. The present study's hypothesis for the divergence from the Hertel classification is that dissections lateral to the humeral head interfere less with medial vascularization, which could reduce the risk of HHO despite the loss of the medial hinge and fragments extending to a head smaller than 8 mm.

Campochiaro et al. observed that Hertel's criteria were not sufficient to determine the risk of developing osteonecrosis, which is similar to the present findings.^5^ However, Hertel has demonstrated that factors including medial hinge loss could increase the risk of osteonecrosis.^10^

Most studies have assessed HHO in patients who underwent osteosynthesis through a deltopectoral approach.^11^^,^^15^ The question of whether the surgical approach (deltopectoral or anterolateral) is related to the development of HHO remains controversial.^16^ A few studies have explored the incidence of HHO after the post-anterolateral approach.^17^ A systematic review by Cochrane was not able to determine whether the approach (deltopectoral or anterolateral) could influence the development of HHO because of the small number of studies related to the anterolateral approach.^6^ Another difficulty is that most studies evaluating the anterolateral approach have used the minimally invasive plate osteosynthesis technique.^18^ Therefore, to our knowledge, this is the first study to correlate the Hertel classification with the risk of HHO after osteosynthesis performed through the anterolateral approach.

The main limitation of the study was the retrospective design, which did not allow comparisons with controls. However, the minimum follow-up period of 1 year after surgery, and the uniform performance of the anterolateral approach, strengthen the present findings.

## Conclusion

Hertel's criteria were not able to predict the risk for HHO after osteosynthesis of proximal humeral fractures performed through the anterolateral approach. The overall prevalence of HHO was 17.9%.

## Conflicts of interest

The authors declare no conflicts of interest.
